# Easy Employment and Crosstalk-Free Detection of Seven Fluorophores in a Widefield Fluorescence Microscope

**DOI:** 10.3390/mps1020020

**Published:** 2018-06-01

**Authors:** Sebastian C. Bhakdi, Ponpan Thaicharoen

**Affiliations:** 1 Department of Pathobiology, Faculty of Science, Mahidol University, Bangkok 10400, Thailand; 2 X-Zell Biotec, Bangkok 10400, Thailand; ponpan.t@x-zell.com

**Keywords:** seven-color immunostaining, rare cell analysis, fluorescence microscopy, immunopathology, slide-based cytometry

## Abstract

Immunofluorescence staining has become an essential tool in pathology and biomedical sciences to identify rare cells, cell–cell interactions, and submicroscopic cellular components. Many experimental settings, however, suffer from the fact that traditional widefield fluorescence microscopy is usually restricted to imaging three or four fluorophores only. Due to a lack of morphological information and a high detection limit, even flow cytometry—which is capable of staining 20 or more fluorophores at the same time—is limited in its applicability, especially in areas such as rare cell detection. Other advanced imaging approaches, such as confocal laser scanning microscopy and imaging flow cytometry, may be addressing these shortcomings, but in turn require sophisticated downstream data processing and high capital outlay. Here, we describe a new method and filter set-up to routinely employ up to seven fluorophores on a traditional widefield fluorescence microscope equipped with a standard high-pressure mercury light source. Quantification of crosstalk between channels and actual seven-color imaging of cancer cells spiked into leukocytes demonstrate that there is no need for digital compensation correction algorithms. Our set-up thus permits a detailed analysis of rare cell populations, co-localization of antigens, and cell morphology in a standard research or routine laboratory setting.

## 1. Introduction

The world of multi-parameter rare cell analysis is changing. In a move to overcome the restrictions of traditional microscopic analyses using fluorophore-labeled antibodies—which are limited to visualizing three to four different cellular markers at a time—new technologies keep emerging that pledge ever-more analytical depth and improved data accessibility. Despite proving resourceful in research settings, most of them are yet to make a notable impact on the clinical routine.

Even flow cytometry, the only analytic tool with multi-parameter capabilities that has achieved significant clinical cut-through to date, comes with a suite of constraints that need improvement—not least its inability to produce the morphological information available via conventional microscopic analyses. Despite being capable of imaging 20 and more fluorophore-labeled antibodies simultaneously, its relatively high detection limit also restricts the use of flow cytometry in rare cell detection [1].

Other emerging multi-parameter approaches to rare cell analysis, such as imaging flow cytometry [2,3], mass spectrometry-based analysis [4,5], and multi-epitope-ligand-cartography (MELC) [6,7], have recently shown potential to address some of these shortcomings, but in turn require relatively expensive additional equipment and sophisticated downstream processing of digitally acquired data—disadvantages that are still holding them back from widespread clinical adoption.

As such, next-generation fluorescence microscopy may be considered the most promising method to analyze rare cell populations in everyday clinical settings, especially given the ongoing development of new fluorophores to label antibodies and the generation of new fluorescent proteins.

One way of multiplexing larger numbers of fluorophores in the microscope is spectral imaging followed by linear unmixing. Since this technology relies on the acquisition of spectral image stacks separated by no more than 2–10 nm, it requires tunable excitation light sources and adjustable emission detection windows. It may also result in very large image data sets and lengthy image acquisition and analysis processes.

One slightly more practical variant using the standard lasers of a confocal microscope as the excitation light source has recently been presented by Gerner et al. [8]. Multi-color laser scanning confocal immunofluorescence microscopy demonstrated five-laser, line-based co-staining of six different fluorophore-labeled antibodies in single-tissue sections when studying murine lymph node dendritic cell subpopulations. However, high spillover—or crosstalk—between emission bands still necessitated significant deconvolution, a computationally intensive processing technique to improve image contrast and resolution. The required compensation algorithms have been limiting the technology’s applicability for daily laboratory use.

In 2014, Eissing et al. went on to combine classical narrow stokes shift fluorescent dyes of the Alexa Fluor series and large stokes shift tandem dyes of the Brilliant Violet series, in turn achieving six-color immunofluorescence microscopy on a confocal microscope with just four lasers and no additional image processing. The tandem’s acceptor dye, however, still exhibited crosstalk into neighboring fluorescence channels [9].

Finally, in 2015, Kijani et al. demonstrated the use of narrow-bandwidth fluorescence filters and standard fluorochromes for six-color immunofluorescence microscopy on a standard widefield fluorescence microscope [10]. The authors reported zero crosstalk into neighboring channels, albeit only judging qualitatively from the acquired images, with no empirical quantification performed.

What is more, Kijani et al.’s staining process mostly involved secondary fluorophore-labeled antibodies, which not only required laborious staining procedures, but also resulted in large variations in staining intensity for each channel. This, in turn, resulted in hugely different image acquisition times, ranging from less than 100 ms up to several seconds per channel.

Progressing further towards the practical routine use of true multi-color microscopy, we now provide a protocol that avoids secondary antibodies or tandem dyes and includes a standardized method to empirically quantify crosstalk between channels. This optimized set-up results in standardized image acquisition times of 50 ms per channel on an automated widefield fluorescence microscope equipped with a standard high-pressure mercury vapor arc-discharge light source. We demonstrate that carefully selected combinations of up to seven different, primary fluorophore-labeled antibodies and microscope fluorescence filter sets enable the fast and direct visualization of several cell subsets, with minimal crosstalk between fluorescence channels and no downstream application of advanced mathematical algorithms.

## 2. Materials and Methods

### 2.1. Cell Lines and Human Blood Samples

A549 lung adenocarcinoma cells were purchased from Cell Line Services (Eppelheim, Germany) and cultured at 37 °C and 5% CO_2_ in DMEM/HAM’S F-12 (1:1), 2 mM l-glutamine supplemented with 10% fetal bovine serum (FBS), 100 U/mL penicillin, and 0.1 mg/mL streptomycin (final concentrations). The cells were harvested with 0.25% trypsin. Blood used to spike experiments was obtained from healthy, voluntary donors in our laboratory.

### 2.2. Erythrocyte Lysis for Isolation of Human Leukocytes

Twenty milliliters of hMX lysis buffer (X-ZELL, Sunnyvale, CA, USA) was added to 5 mL whole blood. The samples were then incubated for 5–7 min (till clear) and washed with 137 mM NaCl, 10 mM phosphate, 2.7 mM KCl, 5 mM EDTA and 1% fetal bovine serum at pH 7.4 (PBS/EDTA/FBS) at 400 *g* for 10 min at room temperature, at the lowest centrifuge acceleration and deceleration. After centrifugation, the leukocytes were re-suspended in 50 µL PBS/EDTA/FBS and counted in a hemocytometer.

### 2.3. Staining of A549 Cells Spiked into Human Leukocytes

The cultured A549 cells were spiked into leukocytes at a ratio of 1:20 before resuspending the mixtures in 700 µL cytocentrifugation buffer (X-ZELL), and spinning them onto gelatin-coated slides (X-ZELL) in one-well concentrators of a Statspin Cytofuge 2 (Beckman Coulter, Brea, CA, USA) for 10 min at 600 rpm at room temperature. The slides were fixed in cryofixation buffer I (X-ZELL) for 15 min at −25 °C and re-hydrated in cryofixation buffer II (X-ZELL) for 20 min at −2.5 °C.

Next, they were mounted on CapGap assemblies (X-ZELL) and inserted in a Cryostainer (X-ZELL). Four microliters of Fc-receptor blocking reagent (Biolegend, San Diego, CA, USA) in 95 µL blocking buffer (X-ZELL) was applied, followed by cocktails of fluorophore-conjugated antibodies in antibody binding buffer as indicated. Antibodies and nuclear dye were as follows: anti-cytokeratin Brilliant Violet 421 (clone: CAM5.2, BD Bioscience, San Jose, CA, USA); anti-CD45 Brilliant Violet 480 (clone: HI30, BD Bioscience); anti-ALDH1 Alexa Fluor 488 (clone: EP1933Y, Abcam, Cambridge, UK); anti-CD3 R-Phycoerythrin (PE) (clone UCHT1, exbio, Prague, Czech Republic); anti-vimentin Alexa Fluor 488, PE and Alexa Fluor 594 (all: clone: EPR3776, Abcam); anti-CD16 PerCp (peridinin- chlorophyll protein complex; clone: 3G8, exbio); and DRAQ5 DNA dye (eBioscience, San Diego, CA, USA). Incubation times were 45 and 60 min for blocking and staining, respectively. After staining, the slides were released from the CapGap clips and coverslipped with 25 µL mounting buffer (MB I) containing DRAQ5 DNA dye.

### 2.4. Widefield Fluorescence Microscopy

**Figure 1:**
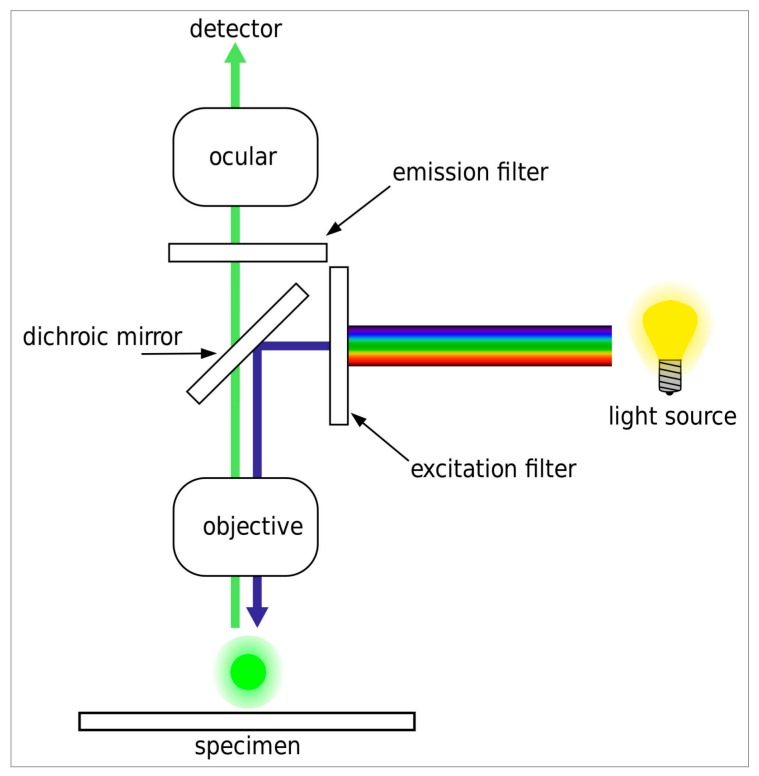
Optical set-up of a standard widefield fluorescence microscope as used in the study. The excitation filter isolates the excitation bandwidth from the light source. The dichroic mirror directs the excitation light through the microscope objective onto the sample. The excitation light excites the fluorophore in the sample, while light emitted from the sample is directed through the dichroic mirror, the emission filter, and the ocular or phototube to the detector (human eye or camera, respectively). Image modified by Henry Mühlpfordt; reproduced according to GNU Free Documentation License.

All slides were analyzed on an automated DM6000B widefield fluorescence microscope equipped with an eight-position filter turret, a 100 W high-pressure mercury vapor arc-discharge light source (Leica, Wetzlar, Germany) and an Orca R2 CCD camera (Hamamatsu, Tokyo, Japan). Images were recorded and analyzed using a 40×/0.75 Fluotar objective (Leica) and MicroManager 1.4 software (MicroManager, San Francisco, CA, USA). The filter sets used for each fluorophore and the optical set-up of the microscope are shown in Table 1 and Figure 1, respectively. The exposure time was set to 50 ms per fluorescence channel at a 1344 × 1024 pixel resolution. For the experimental set-up, we used spectral data in ASCII (American Standard Code for Information Interchange) format and the fluorescence spectra viewer, both from Chroma Technology Corporation (Bellows Falls, VT, USA) [11].

### 2.5. Measuring Integrated Fluorescence Intensity and Determining Crosstalk

To quantify potential crosstalk, we measured the integrated fluorescence density in ImageJ (as described in the software documentation) [12]. After selecting regions of interest (ROI) containing fluorescent events (cells), we used the software’s ROI manager to measure fluorescence density and subtracted the background. The signal intensity of the main channel was normalized to “1”. Signal intensity in the adjacent channels then reflected crosstalk and was expressed as a percentage of the intensity of the main channel.

## 3. Results

### 3.1. Careful Selection of Filters and Fluorophores Avoids Crosstalk in a Seven-Color Widefield Immunofluorescence Microscopy Set-Up

#### 3.1.1. Design of Filter Sets and Fluorophore Panels

In order to detect more than four antigens on cells that have been immunostained on standard laboratory slides—with a widefield immunofluorescence microscope and without the application of compensation correction algorithms [8,13]—we analyzed the absorption and emission spectra of commercially available fluorophores. Fluorophore excitation spectra generally have a slow ascending slope but a steep descending slope. Conversely, emission spectra have a steep ascending slope but a slow descending slope. Based on these observations, we realized that employing narrow bandpass filters in the filter cubes of our widefield microscope should prevent crosstalk between adjacent channels.

Using the Spectra Viewer, we identified Brilliant Violet 421, Brilliant Violet 480, Alexa Fluor 488, PE, PerCp, Alexa Fluor 594, and DRAQ5 as most likely to be separable with optimized filter sets. We optimized our excitation filter sets by selecting narrow bandpass filters at the “foot” of the descending slope of the adjacent shorter-wavelength fluorophore (the “South” fluorophore). Where possible, we also covered the peaks of the UV lamp emission spectrum to provide sufficient excitation energy.

**Figure 2:**
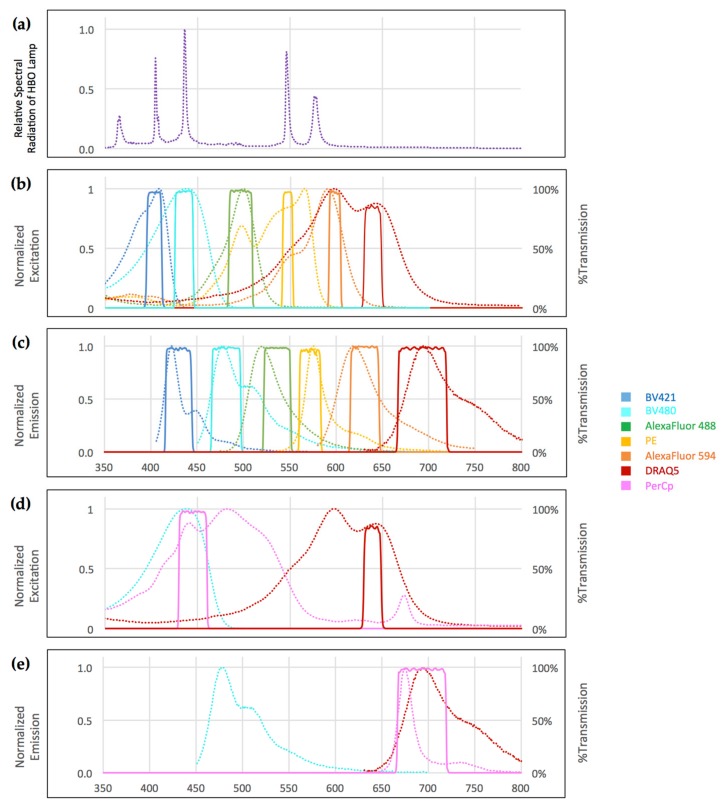
(**a**) Relative spectral radiation of the 100W high-pressure mercury vapor arc-discharge lamp (HBO lamp); (**b**–**e**) Normalized excitation and emission spectra for each fluorophore (dotted lines) and corresponding filter sets (solid lines) for seven-color widefield immunofluorescence microscopy; (**b**,**c**) Minimal overlap was achieved by positioning excitation filter sets at the foot of the descending slope of the preceding fluorophore and emission filters at the foot of ascending slope of the following fluorophore; (**a**,**b**) Mercury lamp radiation peaks at 405, 436, and 546 nm fully coincide with respective excitation filters; (**d**,**e**) Excitation of PerCp and DRAQ5 can be separated relying on the large stokes shift of PerCp while emission spectra overlap. ASCII data downloaded from Chroma Spectra Viewer [11].

Emission filter sets were optimized by selecting narrow bandpass emission filters at the “foot” of the ascending slope of the adjacent longer-wavelength fluorophore (the “North” fluorophore), covering the emission peaks of the target fluorophore where possible. In addition, we chose PerCp as a seventh color, assuming that its very large stokes shift would avoid crosstalk with the other six fluorophores (Figure 2 and Table 1).

#### 3.1.2. Quantification of Crosstalk between Fluorophore Channels

**Figure 3:**
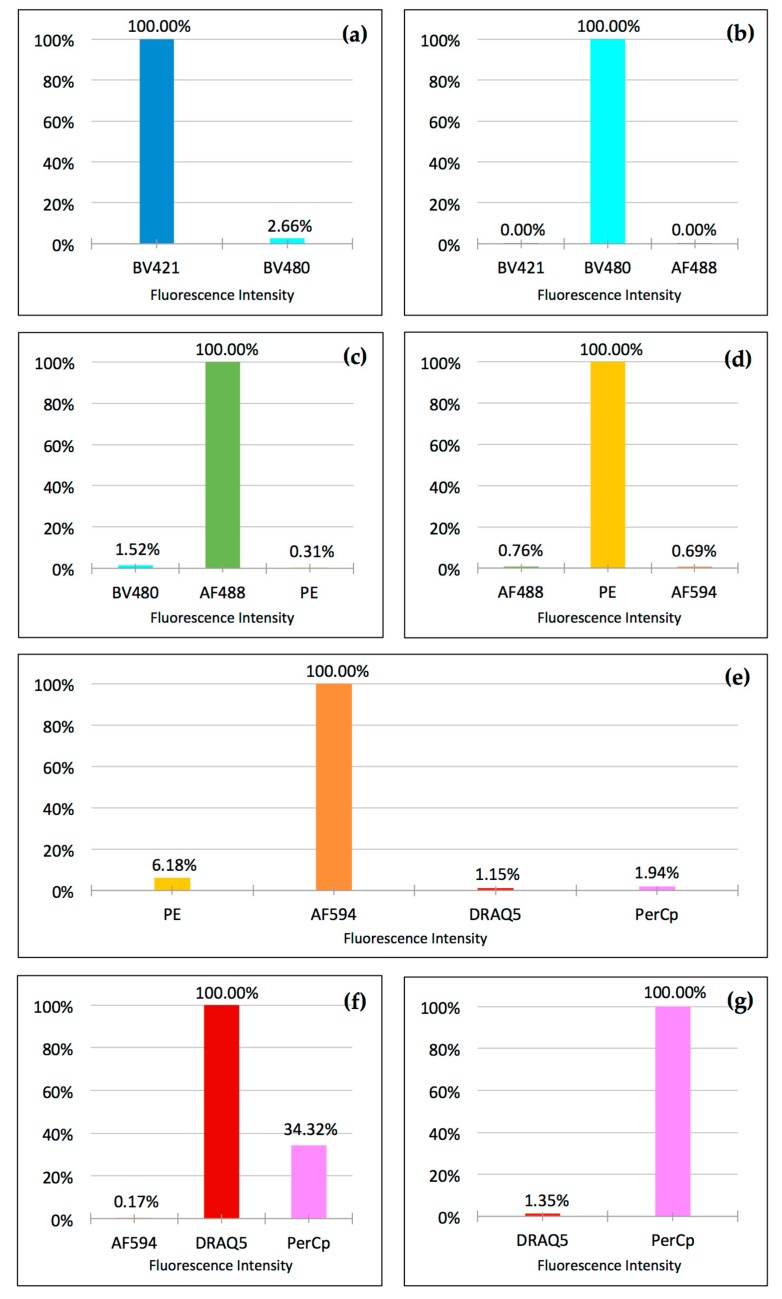
Crosstalk depicted for each fluorophore: Fluorescence intensity of each respective main channel is set to 100%. Crosstalk into adjacent channels is shown as percentage of fluorescence intensity of the main channel. (**a**) Brilliant Violet 421; (**b**) Brilliant Violet 480; (**c**) Alexa Fluor 488; (**d**) PE; (**e**) Alexa Fluor 594; (**f**) DRAQ5; and (**g**) PerCp. Cells were stained on separate slides for each fluorophore and data was acquired on a Leica DM6000B microscope with a 40× objective and 0.63× camera adapter using the 12-bit setting of a monochrome Hamamatsu Orca R2 digital camera. Integrated fluorescence density was determined after the subtraction of background fluorescence in each channel. Data are from at least six cells per channel. Standard deviation was minimal with error bars too small to show. Channels not shown did not exhibit detectable crosstalk.

In set-up experiments, we then immobilized, fixed, and stained cells with only one fluorophore per slide. For this experiment, we chose highly expressed antigens to obtain the brightest-possible signals to maximize any possible crosstalk between channels. In particular, we separately stained A549 cells immobilized on slides with anti-cytokeratin Brilliant Violet 421, anti-vimentin Alexa Fluor 488, PE, and Alexa Fluor 594. White blood cells were separately stained with anti-CD45 Brilliant Violet 480, anti-CD16 PerCp, and DRAQ5. To compensate for the differences in the relative brightness of the different fluorophores (their respective products of extinction coefficient and quantum yield), the potential difference in the fluorophore/antibody ratio present in each of the commercial antibodies used, and the difference in the cellular expression levels of different antigens, we titrated each antibody–fluorophore conjugate to deliver robust visibility in the eyepiece of the microscope. We then determined crosstalk by measuring the integrated fluorescence intensity in channels north and south of the main fluorescence channel, as described in Material and Methods. The optimized fluorophore-filter combinations resulted in minimal crosstalk for all combinations, with the exception of PerCp exhibiting significant crosstalk into the DRAQ5 channel (Figure 3).

### 3.2. Detection of Cancer Cells Spiked into White Blood Cells Using Seven-Color Widefield Immunofluorescence Microscopy

In order to prove our hypothesis, we proceeded to optimize a protocol for the simultaneous staining and rapid detection of six differently fluorophore-labeled antibodies and DNA dye. As a proof of concept, we analyzed immunologically relevant leukocyte and cancer cell antigens. 

#### 3.2.1. Seven-Color Immunostaining

**Figure 4:**
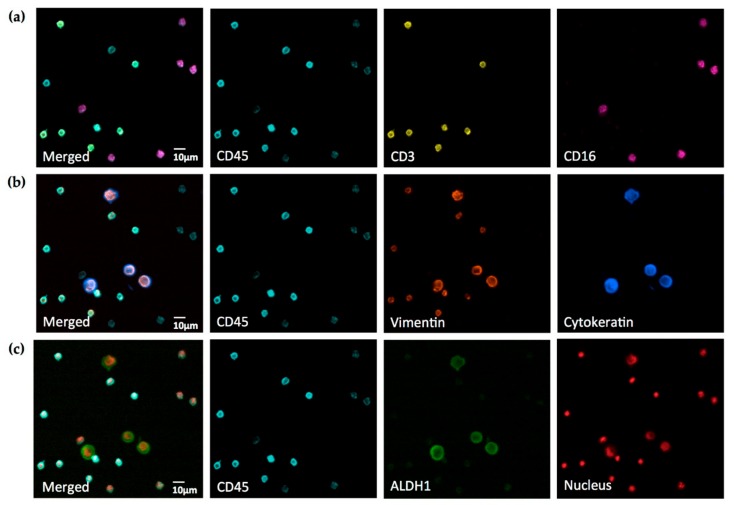
Seven-color widefield immunofluorescence microscopy of A549 cells spiked into human white blood cells. All three rows of pictures are the same field of view. Images of different antigens and nuclear staining, respectively, are merged into the identical image of CD45 staining for clear reference. (**a**) White blood cell membrane antigens; (**b**) cytoskeletal epithelial (cytokeratin) and mesenchymal (vimentin) antigens; (**c**) cytosolic antigen (ALDH1) and nuclear staining. Cells were fixed and stained with anti-cytokeratin Brilliant Violet 421 (1:200), anti-CD45 Brilliant Violet 480 (1:50), anti-ALDH1 Alexa Fluor 488 (1:100), anti-CD3 PE (1:20), anti-vimentin Alexa Fluor 594 (1:500), anti-CD16 PerCp (1:25), and DRAQ5 DNA dye (1:150). Note vimentin^+^CD3^+^CD45^+^ and vimentin^–^CD16^+^CD45^+^ white blood cell populations and vimentin^+^Cytokeratin^+^ALDH1^+^CD45^–^ cancer cells. Cancer cells exhibit markedly different intracellular localization of vimentin and cytokeratin and pronounced aneuploidy. Images were acquired on a Leica DM6000B widefield fluorescence microscope with a 40× Fluotar objective, a 0.63× camera adapter and a Hamamatsu Orca R2 camera. Data is representative for staining of eight different samples on eight different days.

To analyze A549 lung cancer cells spiked into human leukocytes, we used a Leica DM6000B microscope equipped with seven fluorescence cubes, and performed staining with six commercially-available, fluorophore-labeled anti-human antibodies, as well as DRAQ5 DNA dye, as shown in Figure 4. 

In human leukocytes and leukocyte sub-populations, up to five different signals were detected using anti-CD3 PE, anti-CD16 PerCp, anti-CD45 Brilliant Violet 480, anti-vimentin Alexa Fluor 594, and DRAQ5 nuclear dye. At the same time, cancer cells exhibited four different signals from staining with anti-cytokeratin Brilliant Violet 421, anti-ALDH1 AlexaFluor 488, anti-vimentin Alexa Fluor 594, and DRAQ5 nuclear dye.

As expected, all leukocytes stained CD45^+^, while all cancer cells stained CD45^−^. Furthermore, we observed Vimentin expression in CD3^+^CD16^–^CD45^+^ leukocytes, but not in CD3^–^CD16^+^CD45^+^ leukocytes. Cancer cells expressed vimentin, cytokeratin, and ALDH1, and we noted a markedly different intracellular distribution of these proteins, highlighting their different cellular functions. In addition, cancer cells frequently demonstrated atypical, aneuploidic nuclei.

Interestingly, even though the camera was able to measure and “see” crosstalk of DRAQ5 into the PerCp channel and—to a much lower degree—from AlexaFluor 594 into the PE channel, the signal-to-noise ratios in both contaminated channels proved to be sufficiently large to obtain clear images by only adjusting the image brightness.

In summary, highly multiplexed immunostaining on human leukocytes spiked with A549 lung cancer cells illustrates a convenient method to simultaneously stain and rapidly image a set of leukocyte and carcinoma cell antigens on standard laboratory slides.

## 4. Discussion

By imaging human cancer cells spiked into human leukocytes, we were able to demonstrate that up to seven fluorophores can be employed on a standard widefield fluorescence microscope and detected specifically with negligible crosstalk. The resulting seven-color images can be acquired in less than 10 s per seven-channel picture, providing new insights into the co-localization of various cell antigens in human blood. Importantly, the fluorophores employed in this study are standard reagents in flow cytometry and available conjugated to a very wide range of antibodies, which will facilitate the design of suitable antibody panels for most research questions.

The simple staining protocol—which takes advantage of primary fluorophore-conjugated flow-cytometry antibodies—facilitates titration to obtain similar emission brightness in all channels, enabling very fast image acquisition times. The set-up for seven colors also offers additional advantages over the previously published set-up for six-color imaging [10]. In addition, we offer a reproducible way to empirically quantify crosstalk between neighboring channels, which should assist researchers to objectively optimize their own multi-color microscopy set-up.

Compared to other potent cell analysis tools, our approach also enables the observation of several cell subsets and their morphologies—in turn revealing important information about the presence of an antigen at the single cell level, as well as its extracellular, intracellular, or nuclear protein distribution and structure. Characterizing cells in such detail on a single slide can help avoid common interpretation errors, which may occur when analyzing data retrieved via flow cytometry or other methods that do not disclose information regarding cell morphology.

Our experiments also demonstrated that a carefully selected combination of filter sets and fluorophores enables the crosstalk-free visualization of cells fixed and stained on standard slides. The only significant exception was—as expected from our set-up experiments—DRAQ5 talking into the PerCp channel and, to a much lower degree, AlexaFluor 594 talking into the PE channel. However, in actual seven-color staining, the signal-to-noise ratios were sufficiently large to obtain clear images for analysis by only adjusting the image brightness (Figure 4). Interestingly, when using the eyepiece of the microscope, dim crosstalk could be recognized in the PE channel, but not in the PerCp channel. This seemingly contradictory finding can be explained by the fact that the human eye has a much superior dynamic contrast ratio over any modern camera in the visible light spectrum (PE channel), but loses this advantage when approaching the near-infrared spectrum (PerCp channel) [14]. Also, while low-expression antigens may pose challenges by reducing the signal-to-noise ratio, the distinct morphology of nuclei and cellular structures will still enable researchers to distinguish between crosstalk and a specific signal in the case of nuclear staining talking into a membrane antigen channel.

The key to selecting the correct filter set combinations lies in the fluorophores’ distinct slopes of excitation and emission spectra. We were also able to avoid tandem dyes, which tend to exhibit fluorescence bleed-through into the emission channel of the donor-dye and unwanted excitation in the channel of the acceptor dye, as observed in our laboratory and by other authors [9].

It is important to note that the excitation maxima of most fluorophores fall onto, or close to, the emission peaks of classical high-pressure mercury bulbs. In our set-up, the mercury 405 nm peak coincides with the Brilliant Violet 421 excitation maximum, while the 436 nm peak matches with the Brilliant Violet 480 maximum and the PerCp maximum. The 546 nm peak coincides with the PE maximum, and the 579 nm peak is close to the Alexa Fluor 594 maximum. Taking advantage of this observation facilitates the use of very narrow-bandwidth excitation filters. 

In our set-up, the only exceptions were the excitation spectra peaks of Alexa Fluor 488 and DRAQ5, which do not coincide with mercury emission peaks. Therefore, it is advisable to use Alexa Fluor 488 for highly expressed antigens. DRAQ5, on the other hand, binds to DNA stochiometrically, and we found that the concentration can conveniently be up-titrated to give robust visibility in our system. In fact, far-infrared dyes such as AlexaFluor 750 or AlexaFluor 800 could be added to the panel to add an eighth channel. In standard laboratory settings, however, the relatively low spectral radiation of the mercury bulb in this range of the spectrum and limited availability of antibody conjugates somewhat reduce the applicability of these fluorophores.

Taking these physical and physiochemical limitations into account, excitation filters can therefore be chosen with very narrow bandwidths around or close to mercury emission and fluorophore excitation peaks. This minimizes cross-excitation of adjacent fluorophores and—in combination with corresponding narrow-bandwidth emission filters—effectively avoids crosstalk (Figure 2 and Figure 3 and Table 1).

Accordingly, we conclude that if the correct filters and fluorophores are used in combination, there is no need to apply advanced compensation correction algorithms (as recently suggested for another method in histocytometry [8]), which are undesirable as they tend to reduce the image’s signal-to-noise ratio when subtracting the grey values of overlapping emission spectra. In microscopic imaging, a good signal-to-noise ratio is essential to reproducibly detect faint signals of low-expressed antigens and ensure a high sensitivity.

With that in mind, the lack of excitation intensity of the mercury bulb in the 480–510 nm region and the far infrared region somewhat limits the detection of low-expressed antigens at the moment. However, increasing the lamp wattage or switching to high-powered metal-halide or Xenon light sources offering emission spectra that are more consistent could remedy this shortcoming when required.

Compared to flow cytometry—widely considered the most application-rich single-cell characterization technology to emerge over the past 40 years—our approach does not forego crucial information on cell morphology and keeps samples intact for repeat analysis. Cells used in flow cytometry, meanwhile, are considered lost after being analyzed once.

The same is true for the more advanced image flow cytometry approach, which also requires more sophisticated equipment than our widefield microscope-based solution, and with it, on-going maintenance and management by a highly trained specialist.

Other analytic tools, such as confocal immunofluorescence microscopy and MELC [6,7,9]—where multiple fluorescein isothiocyanate (FITC)- or PE-labeled antibodies are used in serial application on tissues or single cells—are also restricted by the need for expensive equipment, and may also require sophisticated downstream processing of digitally acquired data. In a move to maximize clinical utility, our approach successfully avoids all of these constraints.

## 5. Conclusions

In summary, we provide a new, resource- and time-efficient technique to visualize seven fluorophores using a conventional widefield fluorescence microscope equipped with a standard high-pressure mercury light source. Most importantly, our solution is robust and suitable for use in a routine clinical setting—a quality other promising multi-parameter approaches to rare cell analysis are yet to demonstrate.

With advanced image analysis still a considerable bottleneck for many cellular assays, our approach thus allows cells to be distinguished at much greater detail compared to other multi-parameter-based methods, opening up new opportunities for the analysis of rare cell types not routinely detectable with present methods—for example, circulating tumor cells (CTC) or cells obtained from pleural effusion, ascites, or central nervous system (CNS) liquor. Ultimately, seven-color widefield fluorescence microscopy may therefore help us improve our understanding of immunopathological processes and systemic disease.

## Figures and Tables

**Table 1:** Filter sets used for seven-color widefield fluorescence microscopy.

Channel	Fluorophore	Excitation Filter [nm]	Dichroic Mirror [nm]	Emission Filter [nm]
1	Brilliant Violet 421	402/15	412	431/28
2	Brilliant Violet 480	436/20	455	480/30
3	Alexa Fluor 488	495/25	515	537/29
4	PE	546/10	560	572/23
5	Alexa Fluor 594	599/13	612	632/28
6	PerCp	445/30	660	690/50
7	DRAQ5	640/30	660	690/50

PE: R-Phycoerythrin; PerCp: peridinin-chlorophyll protein complex.
